# A stratification system of ferroptosis and iron-metabolism related LncRNAs guides the prediction of the survival of patients with esophageal squamous cell carcinoma

**DOI:** 10.3389/fonc.2022.1010074

**Published:** 2022-09-15

**Authors:** Ren Niu, Fangchao Zhao, Zefang Dong, Zhirong Li, Shujun Li

**Affiliations:** ^1^ Department of Oncology, The Second Hospital of Hebei Medical University, Shijiazhuang, China; ^2^ Department of Thoracic Surgery, The Second Hospital of Hebei Medical University, Shijiazhuang, China; ^3^ Clinical Laboratory Center, The Second Hospital of Hebei Medical University, Shijiazhuang, China

**Keywords:** esophageal squamous cell carcinoma, ferroptosis, lncRNA, prognostic signature, stratification system

## Abstract

Ferroptosis and iron-metabolism have been widely reported to play an important role in cancer. Long non-coding RNAs (lncRNAs) are increasingly recognized as the crucial mediators in the regulation of ferroptosis and iron metabolism. A systematic understanding of ferroptosis and iron-metabolism related lncRNAs (FIRLs) in esophageal squamous cell carcinoma (ESCC) is essential for prognosis prediction. Herein, Pearson’s correlation analysis was carried out between ferroptosis and iron-metabolism-related genes (FIRGs) and all lncRNAs to derive the FIRLs. Based on weighted gene co-expression network exploration (WCGNA), least absolute shrinkage and selection operator (LASSO) regression and Cox regression analysis, a risk stratification system, including 3 FIRLs (LINC01068, TMEM92-AS1, AC243967.2), was established. According to Kaplan-Meier analysis, receiver operating characteristic (ROC) curve analysis, and univariate and multivariate Cox regression analyses, the risk stratification system had excellent predictive ability and clinical relevance. The validity of the established prognostic signature was further examined in TCGA (training set) and GEO (validation set) cohorts. A nomogram with enhanced precision for forecasting OS was set up on basis of the independent prognostic elements. Functional enrichment analysis revealed that three FIRLs took part in various cellular functions and signaling pathways, and the immune status was varied in the high-risk and low-risk groups. In the end, the oncogenic effects of LINC01068 was explored using *in vitro* researches. Overall, a risk stratification system of three FIRLs was found to have significant prognostic value for ESCC and may serve as a ferroptosis-associated therapeutic target in the clinic.

## Introduction

As a common malignant digestive system cancer, esophageal carcinoma was number 8 in morbidity and number 6 in mortality across the world (1). On basis of the National Central Cancer Registry of China (NCCR) statistics, Chinese esophageal cancer patients comprise up to 70% of all esophageal cancer cases worldwide. Esophageal adenocarcinoma and esophageal squamous cell carcinoma (ESCC) are two histopathological subtypes of esophageal cancer. In China, 90% of patients with esophageal cancer are ESCC (2, 3). Standardized surgery is the main treatment for esophageal cancer; however, surgery alone does not often lead to a radical cure for patients with locally advanced esophageal cancer (4). Studies on radiotherapy and chemotherapy, targeted therapy, and biological therapy for the treatment of esophageal cancer have continued over the years; however, the 5-year survival rate of patients with esophageal cancer is less than 20% (5, 6). Hence, new sensitive biomarkers for forecasting the survival of ESCC patients shall be identified as soon as possible.

Iron is elementary for the maintenance of normal roles and homeostasis in cells. Accordingly, an imbalance in iron metabolism is related to the occurrence, growth, and metastasis of cancers closely (7). To be notable, iron metabolism plays double roles in tumor cells (8). In the one aspect, tumor cells proliferate by more depending on iron than normal cells, which is a phenomenon of iron addiction (9). In the other aspect, as iron concentrations increase, cell death will be caused because of accumulated reactive oxygen species and lipid peroxidation outcomes, termed ferroptosis (10, 11). As a necrotic cell death modality, ferroptosis is varied from apoptosis, necrosis, and autophagy in a morphological, biochemical, and genetical way (12). Recently, ferroptosis was revealed to exert various effects on biological regulation and signal transduction paths, resulting in tumor generation and progression (13, 14). Ferroptosis and iron metabolism have also been recognized as hidden preventive or therapeutic measures to cause cancer cell death (15, 16).

Long non-coding RNAs (lncRNAs) have a molecular weight greater than 200 nucleotides. Although lncRNAs account for at least 80% of the human genome, they do not take part in protein translation (17, 18). According to recent studies, the dysregulation of specific lncRNAs is inescapably associated with the ferroptosis process of malignant cancers (19). Further, the upregulation of the lncRNA, NEAT1, was found to potentially regulate ferroptosis sensitivity in non-small cell lung cancer (20). The upregulation of the lncRNA, LINC00336, was also found to inhibit ferroptosis in lung cancer by acting as a contradictive endogenous RNA (21). Nowadays, the effect of lncRNAs on the ferroptosis process of ESCC is unknown.

In the present study, we constructed a risk stratification system, including 3 ferroptosis and iron-metabolism related lncRNAs (FIRLs), and systematically assessed the correlation of the risk stratification system with the prognosis and clinicopathological features of ESCC patients. Thereafter, we established a nomogram that incorporates the FIRL signature and clinical factors to forecast the survival of these patients. Functional enrichment analysis revealed that three FIRLs were involved in various cellular roles and signaling paths, and the immune state was varied in the high-risk and low-risk groups. In the end, the oncogenic effects of LINC01068 were explored using *in vitro* researches, and a new FIRL risk stratification system was developed to enhance the forecast of clinical results in patients with ESCC. To the best of our knowledge, this study firstly constructs and validates a FIRL prognostic signature for ESCC patients.

## Materials and methods

### Datasets and data pre-processing

The RNA-seq transcriptome information and clinical information of ESCC patients were extracted from TCGA database. LncRNAs and protein-coding genes were recognized on basis of annotation documents from the GENCODE database (22). In addition, 296 ferroptosis and iron-metabolism related genes (FIRGs) (**Table S1**), including ferroptosis regulators, ferroptosis markers, ferroptosis pathway, iron uptake and transport, and iron ion homeostasis, were extracted based on previous studies (23). The GSE53624 dataset, which includes RNA-seq information and related survival data of patients suffering from ESCC, was available from the Gene Expression Omnibus (GEO) database. The multi-lncRNA prognostic signature was established with the data from TCGA database as the training cohort while the predictive value of the risk score was determined with the data from GEO as the validation cohort. We performed TPM transformation on the RNA-Seq data of TCGA cohort (FPKM format) and then used the combat method in the”sva”package to remove the batch effect with GEO cohort.

### Identification of FIRLs

Pearson correlation analysis was conducted using the 13,832 lncRNAs and 296 FIRGs identified (*p* < 0.01, correlation coefficient > 0.3). Ultimately, 1,005 FIRLs were screened for follow-up bioinformatics analysis.

### Establishment of the weighted gene co-expression network analysis network

WGCNA is an integrated algorithm for clustering greatly related genes and identifying great modules or core genes related to a given phenotype (24). The present research employed the WGCNA package to set up a gene co-expression network for FIRLs. Briefly, sample clustering was performed using the mean linkage approach to identify and eliminate outlier samples. Thereafter, a suitable soft thresholding power (β = 6) was selected to realize a scale-free topology fitting indicator > 0.9. Outlier samples were eliminated using a suitable cut-off value. As the clustering performed well, a cut-line of 70 was set. Adjacency was then transformed into a topological overlap matrix (TOM) and the corresponding dissimilarity matrix (1-TOM), which was applied to make the gene clustering dendrogram with a minimum module of 50. The merging of greatly similar dynamic modules into larger modules was made at a cutline of 0.6. The associations between the modules and the immune mark were evaluated with Pearson correlation analysis. While identifying the most obvious module, the calculation of gene significance (GS) and module membership (MM) was performed. Key genes were identified as those with GS > 0.7 and MM > 0.7.

### Construction of the risk stratification system

On basis of the clinical information of ESCC cases in TCGA, univariate Cox regression for FIRLs in the hub module was adopted for the identification of FIRLs associated with total survival for risk stratification system establishment. LncRNAs with a P value less than 0.01 were regarded as obvious prognostic signature. To avoid the collinearity of high-dimensional transcriptome data, the “glmnet” package was employed for least absolute shrinkage and selection operator (LASSO) regression. Finally, the best risk stratification system on basis of FIRLs was established using multivariate Cox regression. In particular, the risk score was determined for ESCC cases using the formula below: risk score = (lncRNA 1 expression × coefficient) + (lncRNA 2 expression × coefficient) + … + (lncRNA n expression × coefficient). According to the cut-off value of the risk score, ESCC patients in TCGA and GEO cohorts were fallen into high-risk or low-risk groups.

### Assessment of the clinical benefit

Kaplan-Meier analysis and area under the ROC curves were used for the evaluation of the survival benefit, while independent prognostic factors for patients with ESCC were identified by performing univariate and multivariate Cox regression analyses. On basis of the median value of the risk score and the total survival among various groups were compared through Kaplan-Meier analysis with the log-rank test. Thereafter, the predictive precision of the FIRL signatures was evaluated by conducting a time-dependent ROC curve analysis with “survivalROC” R package. To confirm the value of the stratification system for evaluating the prognosis of ESCC patients, we combined clinical variables and performed univariate and multivariate Cox regression analyses in TCGA and GEO cohorts, respectively. To confirm the prognostic value of the stratification system for evaluating different clinical subtypes of ESCC patients, we combined patients from the GEO and TCGA cohorts into different clinical subtypes to explore the association between risk scores and clinical subgroups. A stratification system for predicting survival was also assessed using PCA, AUC, and decision curve analysis (DCA) curve to weigh the clinical practicability.

### Visualization of the risk stratification system

Multivariate Cox regression analysis was adopted for the estimation of hazard ratios (HRs) and 95% confidence intervals (CIs). The “rms” R packages were employed to formulate a nomogram. The establishment of a prognostic nomogram included all independent prognostic elements recognized by multivariate Cox regression analysis to determine the potential 1-, 3-, and 5-OS of ESCC. The predictive ability of the nomogram was evaluated with AUC and calibration curve. A stratification system for predicting survival was also assessed using PCA, AUC, and DCA curve to weigh the clinical practicability.

### Construction of a potential regulatory network and functional analysis

For exploring the hidden biological processes involving the 3 FIRLs, we identified 49 possible upstream regulated FIRGs through Pearson correlation analysis. Thereafter, gene enrichment analysis was performed with differentially expressed FIRGs using “ggplot2” and “clusterProfiler” packages in R software.

### Immune landscape analysis

We used single-sample gene set enrichment analysis (ssGSEA) (25) to conduct immune landscape analysis and then calculate the scores of infiltrating immune cells to evaluate the activity of immune-related pathways.

### Vitro assays

In this study, we used cell culture, transfection, CCK-8, and qRT-PCR as *in vitro* assays. Human normal esophageal epithelium cells (HET-1A) and ESCC cell lines (Eca109, TE-1, and KYSE-150) were purchased from the Shanghai Cell Institute Country Cell Bank. All cell were cultured in RPMI 1640 medium with 10% fetal bovine serum (FBS) and 1% Penicillin-Streptomycin, and maintained in a humidified incubator at 37°C, 5% CO_2_. Medium, FBS and Penicillin-Streptomycin were purchased from Corning. Guangzhou Ribobio Co., Ltd generated and annealed small-interfering RNA (si-RNA-1/2/3) oligos for LINC01068 and a general negative control. Following the manufacturer’s procedure, the transfection of each siRNA duplex into cells was made with Lipofectamine^®^ 2000 (Invitrogen, Carlsbad, CA, USA). RNA samples from the cultured cells were extracted using the FastPure^®^ Cell/Tissue Total RNA Isolation Kit V2 (Vazyme, Nanjing, China). The concentration and purification of RNA were detected by the Nanodrop 2000 Spectrophotometer (Thermo Scientific, USA). Cell proliferation was monitored using the CCK-8 kit (Dojindo, USA). Details of these methods are provided elsewhere (26). Meanwhile, a total of 10 tumor tissue samples and nearby normal esophageal tissue samples were obtained from ESCC patients who underwent tumor resection. In previous studies, we have cryopreserved cDNA in liquid nitrogen container. Therefore, lncRNAs expression of clinical samples was validated according to previous methods (27, 28).

## Results

### Identification of hub module invovled in disease progression

**Figure 1:**
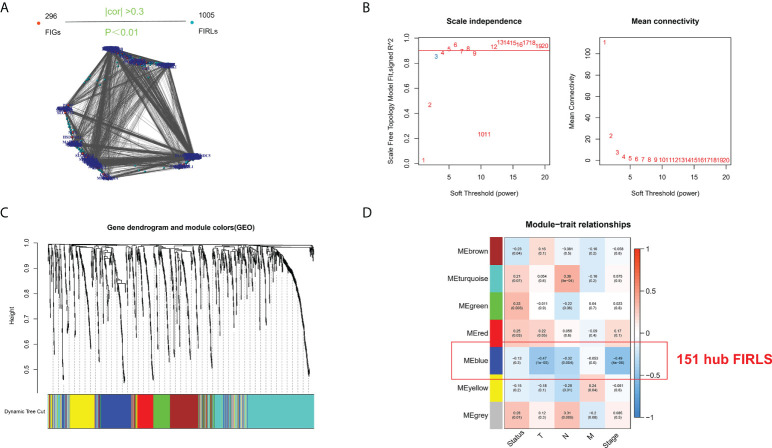
Screening of survival-related lncRNAs by WGCNA. **(A)** Identification of FIRLs using Pearson correlation analysis. **(B)** Soft power in WGCNA. **(C)** Clustering and merging of the co-expression modules. **(D)** Association heatmap of module genes and clinical features. Red means positive association, and blue refers to negative association. Correlation grows as the color darkens.

Based on previous literature, we collected 296 FIRGs (**Table S1**). For TCGA cohort, Pearson correlation analysis was performed using the 296 genes and all annotated lncRNAs. Ultimately, 1005 FIRLs were we identified (**Figure 1A**). The clustering of each sample was good, and only one outlier sample was eliminated (the cutting line was 500). Topological calculation was then performed with a soft threshold value of 1 to 20, and an optimal soft threshold value of 4 (**Figure 1B**). According to the soft threshold, the relationship matrix was finally converted into a TOM, and the related modules were classified according to the TOM. The number of genes in each module was not less than 50, and the shear height of gene modules was 0.6 (**Figure 1C**). By using Pearson correlation analysis to decide the correlation between the modules and clinical traits, seven modules were identified. Of note, the blue module had the strongest correlation with pathological staging and survival status. Accordingly, this module was recognized as the core module in ESCC patients (**Figure 1D**). Finally, the 151 FIRLs in the module were found to be associated with the occurrence of ESCC closely (**Table S2**).

### Construction of risk stratification system

**Figure 2:**
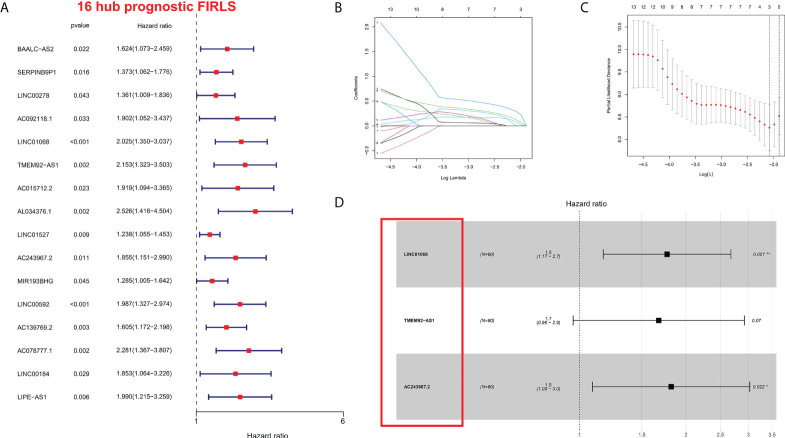
Construction of the risk stratification system. **(A)** The FIRLs that significantly correlated with survival were identified by univariate analysis. **(B, C)** LASSO-validation. **(D)** Forest plot of hazard ratios showing the prognostic value of the 3 FIRLs. *P < 0.05, **P < 0.01.

To identify survival-related FIRLs, univariate Cox analyses were performed using 151 FIRLs in the blue module. Finally, 16 FIRLs were screened for subsequent analyses (**Figure 2A**). For further decreasing the number of genes in the signature, the subjection of 16 FIRLs to LASSO regression analysis was performed (**Figures 2B, C**). Thereafter, three FIRLs from LASSO were retrieved and subjected to multivariate Cox regression analysis to develop a risk stratification system (**Figure 2D**; **Table 1**). The calculation of the risk score of ESCC patients was made below: risk score = 0.5697×LINC01068 + 0.5154 × TMEM92-AS1 + 0.5964 × AC243967.2).

**Table 1:** The optimal prognostic risk stratification system of 3 lncRNAs by multivariate Cox regression analysis.

LncRNA	coef	HR	HR.95L	HR.95H	P-value
LINC01068	0.5697	1.7678	1.1665	2.6788	0.0072
TMEM92-AS1	0.5154	1.674	0.9593	2.9223	0.0697
AC243967.2	0.5964	1.8157	1.0883	3.0292	0.0224

### Clinical benefits of the risk stratification system

**Figure 3:**
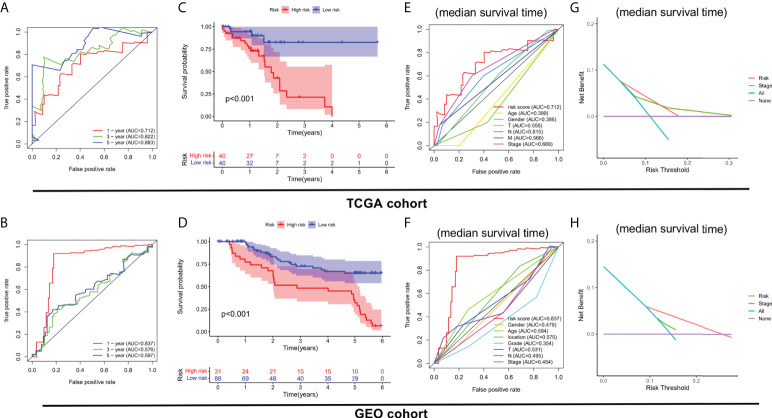
Clinical benefits of the risk stratification system. **(A, B)** Time-dependent ROC curves. **(C, D)** Kaplan-Meier analysis of high- and low-risk patients. **(E, F)** ROC curve analysis revealed the prognostic accuracy of risk mark and clinicopathological coefficients. **(G, H)** Decision curve analysis (DCA).

On basis of the cut-off value of risk scores, ESCC patients from the TCGA cohort were divided into two risk groups: high-risk (*n* = 40) and low-risk (*n* = 40). Using the same cut-off value, ESCC patients in the GEO cohort were fallen into high-risk (*n* = 31) and low-risk (*n* = 88) groups. As shown in **Figure 3A**, the AUCs of the 3 FIRL risk stratification system performed with TCGA cohort were 0.712, 0.822, and 0.883 at 1, 3, and 5 years. In addition, the 1-year survival prediction in the GEO cohort showed good results (**Figure 3B**). The association between the risk mark and prognosis of ESCC patients was explored with the Kaplan-Meier method and log-rank tests. Patients in the high-risk group were found to have a lower survival rate than those in the low-risk group (*P* < 0.001) (**Figures 3C, D**). Furthermore, in the prediction of median survival time, ROC curve analysis revealed that the risk mark showed better predictive performance than the other clinicopathological features (**Figures 3E, F**). DCA also suggested that in actual clinical applications, this risk score had a better value than traditional pathological staging (**Figures 3G, H**).

### Risk stratification system is an independent prognostic element for ESCC patients

**Figure 4:**
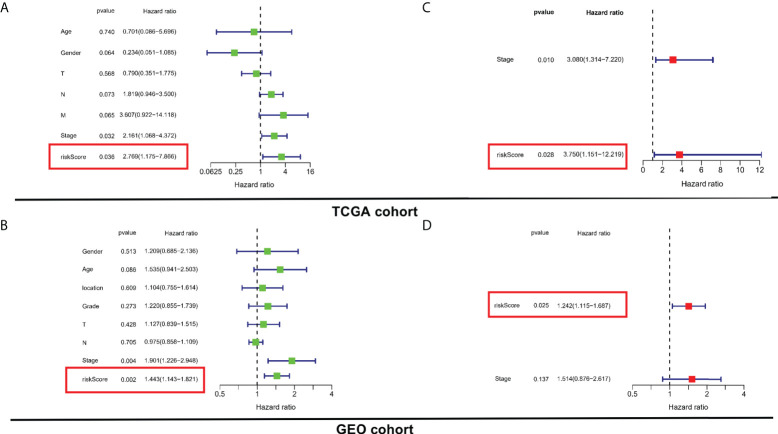
Evaluation of the prognostic values of risk stratification. **(A, C)** Univariate and multivariate Cox regression analyses of the risk scores in TCGA. **(B, D)** Univariate and multivariate Cox regression analyses of the risk scores in GEO.

For determining whether the risk score was an independent prognostic element for ESCC patients, univariate and multivariate Cox regression analyses were conducted using the clinical features and risk score. Based on the outcomes of univariate Cox regression analysis, the risk score was greatly related to OS in both TCGA and GEO cohorts (TCGA cohort: HR = 2.769, 95% CI = 1.175-7.866, *p* = 0.036; GEO cohort: HR = 1.443, 95% CI 1.143-1.821, *p* = 0.002) (**Figures 4A, B**). After the modification for other confounders, the risk score was still an independent predictor of OS in multivariate Cox regression analysis (TCGA cohort: HR = 3.750, 95% CI = 1.151-12.219, *p* = 0.028; GEO cohort: HR = 1.242, 95% CI = 1.115-1.687, *p* = 0.025; **Figures 4C, D**).

### Subgroup analysis of the risk stratification system in the total cohort

**Figure 5:**
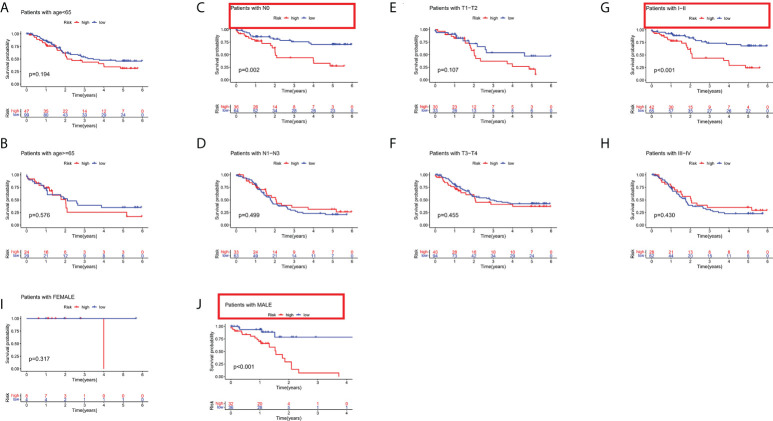
Subgroup analysis based on different clinical characteristics of the risk stratification system. **(A)** age ≤ 65, **(B)** age > 65, **(C)** NO, **(D)** N1-N3, **(E)** T1-T2, **(F)** T3-T4, **(G)** stage I-II, **(H)** stage III-IV, **(I)** female, and **(J)** male.

To determine the prognostic value of the risk stratification system for ESCC patients based on different clinical characteristics, subgroups were derived on basis of age (≤65 vs. >65 years), sex (male vs. female), clinical phase (I-II vs. III-IV), T phase (T0-T2 vs. T3-T4), and N phase (N0 vs. N1-N3). The results indicated that the risk stratification system has prognostic significance between high and low risk patients for N0, I-II, and male subgroups. Patients in the high-risk group shown significantly poorer OS than patients in the low-risk group (**Figures 5A–J**). In sum, these results testify that the risk stratification system exerts critical roles in determining the prognosis of ESCC patients.

### Survival analysis and clinical correlation analysis of FIRLs in the risk stratification system

**Figure 6:**
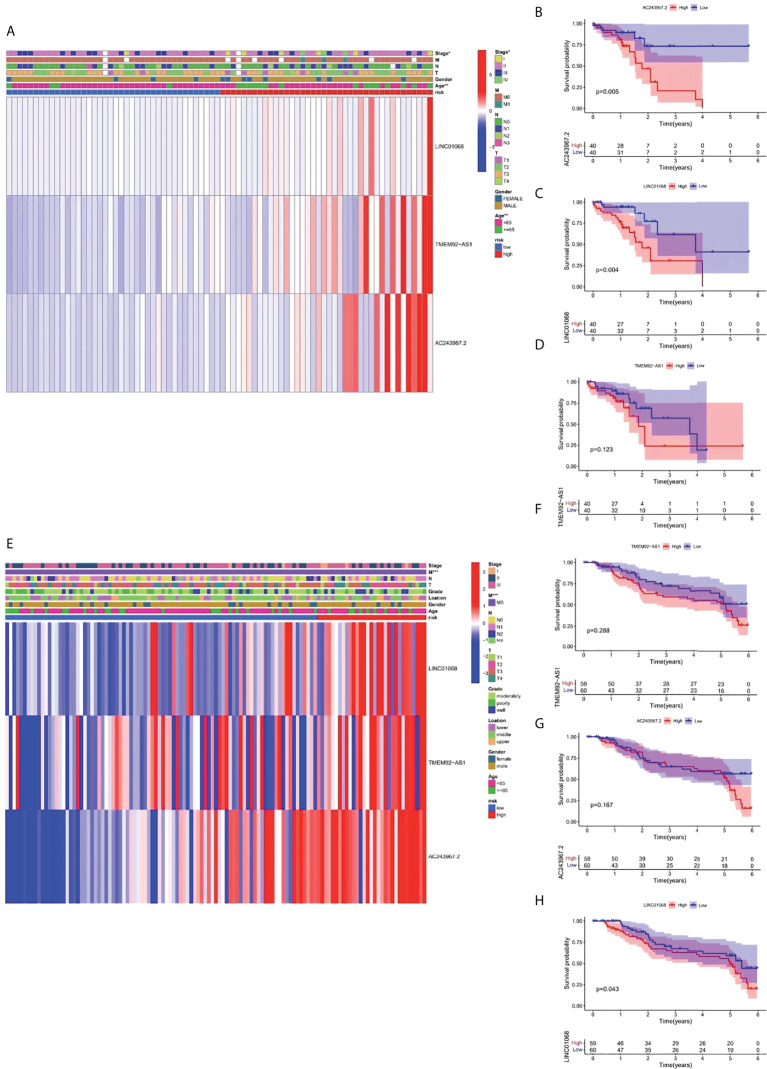
Survival analysis and clinical correlation analysis of FIRLs in risk stratification. **(A)** A composite heat map containing clinical information and expression of 3 FIRLs in TCGA cohort. **(B-D)** Survival analysis of FIRLs participating in risk stratification in TCGA cohort. **(E)** A composite heat map containing clinical information and expression of 3 FIRLs in the GEO cohort. **(F**–**H**) Survival analysis of FIRLs participating in risk stratification in the GEO cohort. *P < 0.05, **P < 0.01, ***P < 0.001.

For further exploring the association between the risk stratification system and clinical parameters, we constructed two composite heat maps for patients from TCGA (**Figure 6A**) and GEO (**Figure 6E**) cohorts. A heat map could display the risk scores, clinicopathological parameters, and FIRL expression for each group. A survival analysis of FIRLs participating in the risk stratification system was also performed. Based on the results, AC243967.2 and LINC01068 were identified as high-risk factors for ESCC patients (high expression of AC243967.2 and LINC01068 was related to poor survival rate of ESCC patients) (*P* < 0.05) in TCGA cohort (**Figures 6B–D**). In the GEO cohort, LINC01068 was proved to be a high risk factor for ESCC (**Figures 6F–H**).

### Construction and verification of nomogram based on the risk stratification system

**Figure 7:**
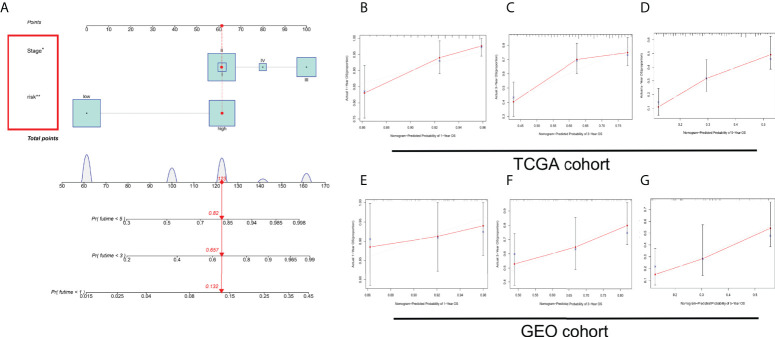
Establishment and verification of a nomogram. **(A)** Nomogram for forecasting the total survival (OS) of patients with ESCC at 1, 3, and 5 years. Calibration curves of nomogram for OS forecast at 1, 3, and 5 years in **(B–D)** TCGA and **(E–G)** GEO cohorts. *P < 0.05, **P < 0.01.

The OS of patients with ESCC was predicted by establishing a nomogram on basis of independent predictive elements originated from a multivariate Cox risk regression model (**Figure 7A**). According to the prediction model calibration curve, consistent predicted and actual survival rates for the training and validation sets were revealed (**Figures 7B–G**).

### Regulatory network of the potential biological functions of 3 FIRLs

**Figure 8:**
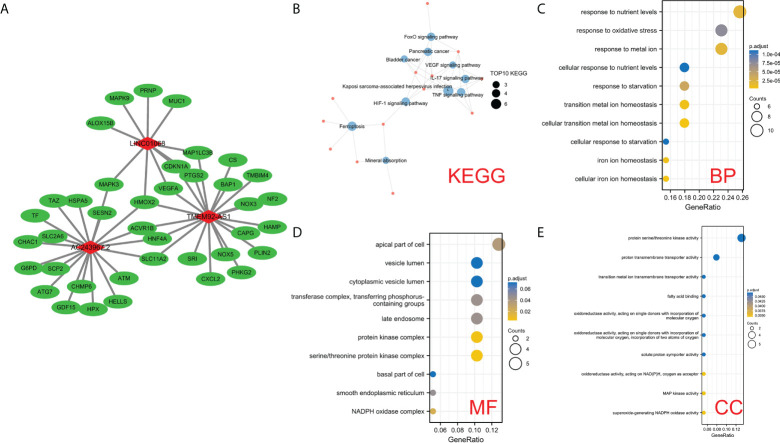
Analysis of the potential functions of 3 FIRLs. **(A)** Regulation network diagram of the 49 upstream coding FIRGs of 3 FIRLs. **(B)** KEGG enrichment analysis of 49 upstream FIRGs. **(C–E)** GO enrichment analysis of 49 upstream FIRGs.

To explore the potential biological processes involving the three FIRLs, 49 possible upstream regulated FIRGs were identified through Pearson correlation analysis (**Figure 8A**). The analyses of GO functional enrichment and KEGG pathway enrichment were conducted on the 49 FIRGs. On basis of the outcomes of KEGG analysis, in addition to ferroptosis, 49 FIRGs were mainly enriched in the IL-17 signaling pathway, HIF-1 signaling pathway, VEGF signaling pathway, and TNF signaling pathway (**Figure 8B**). Further, GO analysis results indicated that in addition to iron death, iron metabolism, and other related processes, the 49 FIRGs were related to DNA damage response and signal transduction by p53 class mediator processes (**Figures 8C–E**).

### Immunity analyses

**Figure 9:**
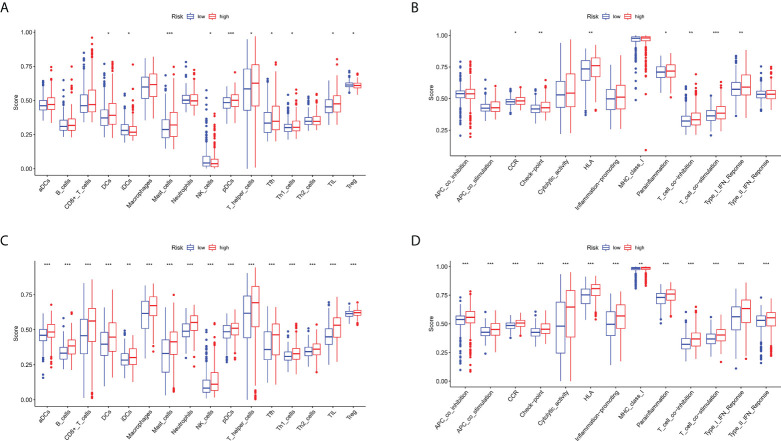
Comparison of the ssGSEA scores for immune cells and immune pathways. **(A, B)** Comparison of the enrichment scores of 16 types of immune cells and 13 immune-related pathways between low- (green box) and high-risk (red box) group in the TCGA cohort. **(C, D)** Comparison of the enrichment scores of 16 types of immune cells and 13 immune-related pathways between low- (green box) and high-risk (red box) group in the GEO cohort. *P < 0.05, **P < 0.01, ***P < 0.001.

Given that ferroptosis and iron-metabolism plays a critical role in the immune processes in human cells, especially in the tumor microenvironment, we compared the enrichment scores of 16 types of immune cells and the activity of 13 immune-related pathways between the low- and high-risk groups in both the TCGA and GEO cohorts by employing ssGSEA. In the TCGA cohort (**Figure 9A**), the high-risk group generally had high levels of infiltration of immune cells, especially of DCs, mast cells, pDCs, T helper (Th) cells (Tfh and Th1 cells), and tumour-infiltrating lymphocytes (TILs), than the low-risk group. In addition, patients from the high-risk group had significantly higher activity of chemotactic cytokines receptors (CCR) pathway, check-point, human leukocyte antigen (HLA) pathway, parainflammation, T cell co-inhibition, T cell co-stimulation, and type I IFN response pathway compared to patients in low-risk group (**Figure 9B**). When assessing the immune status in the GEO cohort, better conclusions were drawn. The infiltration level of 16 immune cells was higher in the high-risk group than in the low-risk group. Thirteen immune-related pathways showed higher activity in the high-risk group than in the low-risk group (**Figures 9C, D**).

### *In vitro* assays for validation

**Figure 10:**
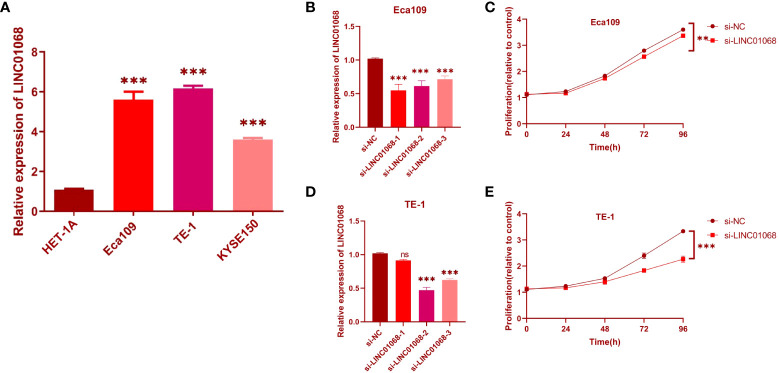
Effects of inhibiting the expression of LINC01068 on cell proliferation. **(A)** The upregulation of LINC01068 was displayed in Eca109, TE-1, and KYSE-150 cells compared to HET-1A based on qRT-PCR. **(B, C)** The expression of LINC01068 was downregulated in TE-1 and Eca109 cells by si-RNAs. **(D, E)** TE-1 and Eca109 cell proliferation after measuring anti-LINC01068 siRNA transfection with CCK-8 assays. ns, not significant, **P < 0.01, ***P < 0.001.

To further validate the bioinformatics results, the expression level of LINC01068 mRNA in ESCC cell lines was detected. It was found that the expression of LINC01068 is upregulated in ESCC cell lines by comparing with the normal cell line, as shown in **Figure 10A**. In addition, si-LINC01068 and si-NC were transfected into Eca109 and TE-1 cells, respectively, and qRT-PCR was adopted for the detection of the expression of LINC01068. LINC01068 expression was downregulated in ESCC cell lines after transfection, as shown in **Figures 10B, C**. Similarly, the CCK-8 assays revealed that ESCC cell proliferation was inhibited after transfection with LINC01068, as shown in **Figures 10D, E**. Meanwhile, qRT-PCR was used to detect the expression of the three lncRNAs in 10 pairs of tissues (**Figure S1**). The results were consistent with the prediction results in public databases. Tumor tissues showed obviously higher expression levels than the normal esophageal tissues.

## Discussion

The transformation of next-generation sequencing has been performed for prognosis of cancer. In clinical routines, the prognosis of cancer patients cannot be adequately predicted with the conventional staging system. Biomarkers associated with tumor diagnosis and prognosis are thus urgently needed (29, 30). Due to the disturbances in iron metabolism, overmuch intracellular iron storage was caused with ferroptosis induced (31). As a hallmark of tumors, Ferroptosis is greatly related to the prognosis of cancer patients (32). Because of the significant effect of ferroptosis and iron metabolism on cancer, remarkable attention has been paid to its associated lncRNAs (33).

To the best of our knowledge, this research firstly identifies and analyzes prognostic FIRLs in ESCC in a comprehensive way. On basis of previous studies, we collected 296 FIRGs. In TCGA cohort, Pearson correlation analysis of 296 genes and all annotated lncRNAs was performed, and 1005 FIRLs were identified. Through WGCNA, 151 core FIRLs were identified in the blue module, and a risk stratification system comprising 3 FIRLs (LINC01068, TMEM92-AS1, and AC243967.2) was established by integrating LASSO regression and Cox regression analyses. The assignment of all patients to high- and low-risk groups was performed on basis of risk scores. On basis of Kaplan-Meier curve analysis, high-risk groups were related to dismal OS by comparing with low-risk groups. The ROC curve indicates the excellent performance of our risk stratification system. The AUCs of the ROC plots for one-, three-, and five-year OS in TCGA cohort were 0.712, 0.822, and 0.883. In addition, the stratification system for predicting survival was assessed by PCA, AUC, and DCA curve to weigh the clinical practicability. Based on the results, our risk signature consistently realized good predictive value by comparing with other risk prognostic signatures published for ESCC. The GEO cohort was adopted to verify the established prognostic signature. Moreover, other clinicopathological features and prognostic signatures were combined for Cox analysis, which ultimately verified that the constructed risk stratification system may be used as an independent prognostic element for ESCC patients. Herein, while establishing a nomogram, whether the nomogram was precise at predicting one-, three-, and five-year OS was determined with calibration plots. Altogether, our findings indicate that the risk stratification system could be a high-class predictor relative to the conventional clinical indicator. To determine the potential biological processes involving the 3 FIRLs, we identified 49 possible upstream regulated FIRGs through Pearson correlation analysis, and further conducted functional enrichment analysis with these genes to dig the potential biological pathways.

Tumor-related immune responses play important roles in cell infiltration and metastasis in the tumor microenvironment, whereas ferroptosis and lncRNAs play key regulatory roles in tumor-related immune responses (34, 35). Notably, the complex interplay between ferroptosis-related lncRNAs and the tumor microenvironment not only plays a pivotal role in tumor development but also has significant effects on immunotherapeutic efficacy and overall survival (36). In the TCGA cohort, by immune infiltration analysis, the high-risk group generally had high levels of infiltration of immune cells, especially of DCs, mast cells, pDCs, T helper (Th) cells (Tfh and Th1 cells), and tumour-infiltrating lymphocytes (TILs), than the low-risk group. A functional enrichment analysis indicated that patients with high-risk scores had higher activity of chemotactic cytokines receptors (CCR) pathway, check-point, human leukocyte antigen (HLA) pathway, parainflammation, T cell co-inhibition, T cell co-stimulation, and type I IFN response pathway compared to patients with low-risk scores. When assessing the immune status in the GEO cohort, better conclusions were drawn. The infiltration level of 16 immune cells was higher in the high-risk group than in the low-risk group. 13 immune-related pathways showed higher activity in the high-risk group than in the low-risk group. The above results confirm that the roles of ferroptosis-related lncRNAs in the regulation of tumor immune infiltration. Since our results link FIRLs to immune infiltration in ESCC, these ferroptosis-related lncRNAs may be targets for immunotherapy.

In the end, the association between LINC01068 and ESCC progression was determined. The inhibition of LINC01068 inhibited the cell viability and migration of Eca109 and TE-1 cells, which further verified the carcinogenic effect of LINC01068 on digestive system neoplasms.

This study had some limitations. First, the FIRL risk stratification system was constructed and validated using a public database. However, the use of prospective, multicenter, real-world data for the assessment of the clinical utility of this system would be more ideal. Second, the association between FIRLs and anti-tumor immunity was preliminarily revealed by our research. Therefore, it is necessary to further dig the hidden mechanisms. Final, the signaling pathways involved in FIRLs were only preliminarily explored. Accordingly, the specific mechanism of FIRLs in ESCC and their association with ferroptosis are not completely acknowledged. More studies are thus needed to validate our findings.

In summary, this study fills a gap regarding the use of FIRLs for the prognostic forecast of ESCC. The prognostic FIRLs derived in our research displayed robust capacity at forecasting the survival results of ESCC patients and were related to the immune landscape of the ESCC microenvironment. The risk stratification system based on FIRLs could serve as a reliable tool for forecasting the survival of patients with ESCC.

## Data availability statement

The original contributions presented in the study are included in the article/**Supplementary Materials**, further inquiries can be directed to the corresponding author/s. Complete dataset can be found here - https://www.jianguoyun.com/p/DdBumiQQzc6eChiA_s4EIAA

## Ethics statement

The studies involving human participants were reviewed and approved by The Ethics Committee of the Second Hospital of Hebei Medical University. The patients/participants provided their written informed consent to participate in this study.

## Author contributions

RN conceived, designed, and wrote the manuscript. RN and FZ assisted in specimen collection and performed experimental work. ZD and ZL were responsible for the data analysis and figures plotted. SL helped with manuscript and data review. All authors contributed to the article and approved the submitted version.

## Funding

This study was supported by Science and Technology Plan Project of Hebei Province (No.223777106D) and Hebei Provincial Government sponsored the project of training excellent talents in clinical medicine (No. 303-2022-27-37).

## Conflict of interest

The authors declare that the research was conducted in the absence of any commercial or financial relationships that could be construed as a potential conflict of interest.

## Publisher’s note

All claims expressed in this article are solely those of the authors and do not necessarily represent those of their affiliated organizations, or those of the publisher, the editors and the reviewers. Any product that may be evaluated in this article, or claim that may be made by its manufacturer, is not guaranteed or endorsed by the publisher.

## Supplementary material

Supplementary Figure 1 The expression of 3 lncRNAs between tumor tissues and normal esophageal tissues.
