# Supplementary material for: A stratification system of ferroptosis and iron-metabolism related LncRNAs guides the prediction of the survival of patients with esophageal squamous cell carcinoma
Source: Front Oncol. 2022 Sep 15;12:1010074. doi: 10.3389/fonc.2022.1010074 (PMC9520776; doi:10.3389/fonc.2022.1010074)
Supplement: Supplementary file 2 [file Table_2.docx]

**Table S2. 151 Ferroptosis and iron-metabolism related lncRNAs.**

| LINC00887 |
| --- |
| BAALC-AS2 |
| CBR3-AS1 |
| AL391845.1 |
| PINK1-AS |
| AC093865.1 |
| AL050341.2 |
| AL022315.1 |
| ARNTL2-AS1 |
| AC082651.3 |
| LINC01338 |
| LINC01214 |
| LINC00960 |
| SERPINB9P1 |
| AC244205.1 |
| LINC02257 |
| LINC02487 |
| LINC02012 |
| AP000866.2 |
| MIR210HG |
| LINC01206 |
| AC093904.4 |
| LINC00278 |
| AL603839.3 |
| AC027117.2 |
| AL358394.1 |
| AC005532.1 |
| DLX2-DT |
| ACBD3-AS1 |
| AL158212.3 |
| LINC02303 |
| LINC02538 |
| DOCK9-DT |
| AC093159.1 |
| ADIRF-AS1 |
| AL451042.2 |
| AC092118.1 |
| LINC02036 |
| AC108134.1 |
| TDRG1 |
| LINC01559 |
| FALEC |
| LINC01251 |
| LINC00265 |
| LINC02100 |
| AC004847.1 |
| AC091057.1 |
| AL139393.1 |
| BACH1-IT1 |
| CCDC183-AS1 |
| AC034213.1 |
| AP001347.1 |
| U62317.1 |
| AC006372.1 |
| LINC01068 |
| IL12A-AS1 |
| AC097359.2 |
| TMEM92-AS1 |
| AC068707.1 |
| LINC01602 |
| AL079303.1 |
| LINC01001 |
| LINC02593 |
| AC080129.1 |
| LINC02568 |
| LINC01426 |
| LINC00330 |
| UBE2R2-AS1 |
| AL122035.1 |
| AC015712.2 |
| HOTAIRM1 |
| AL034376.1 |
| LINC01527 |
| AC243967.2 |
| MIR193BHG |
| ARHGAP5-AS1 |
| CYP4F26P |
| AC006372.2 |
| LINC01589 |
| AC008760.1 |
| AC134312.5 |
| TTTY14 |
| BX322234.1 |
| FLG-AS1 |
| LINC02228 |
| AL161785.1 |
| AL033397.1 |
| STXBP5-AS1 |
| AC104695.2 |
| AF279873.3 |
| AC069287.3 |
| AC018647.2 |
| AC147067.1 |
| FLVCR1-DT |
| AC004990.1 |
| C17orf77 |
| AL357033.1 |
| AL359979.1 |
| LINC02137 |
| AC083841.1 |
| LINC01705 |
| PLA2G4E-AS1 |
| B4GALT1-AS1 |
| AP006621.4 |
| AC018450.1 |
| AC105460.2 |
| IGF2BP2-AS1 |
| LINC00592 |
| AC104083.1 |
| AC109454.3 |
| AC245060.2 |
| FBXL19-AS1 |
| AC139769.2 |
| LINC02310 |
| AC078777.1 |
| EPB41L4A-DT |
| AL031985.3 |
| SSTR5-AS1 |
| AC025048.4 |
| AC011483.2 |
| LINC00957 |
| LINC00886 |
| SOX21-AS1 |
| LUCAT1 |
| CALML3-AS1 |
| GRPEL2-AS1 |
| AL353740.1 |
| RBPMS-AS1 |
| CFAP58-DT |
| LINC00184 |
| ARHGAP27P1-BPTFP1-KPNA2P3 |
| LINC00520 |
| AL355916.1 |
| LIPE-AS1 |
| AL138963.1 |
| AL136982.6 |
| DLX6-AS1 |
| AC010307.2 |
| LINC01094 |
| HCG22 |
| AC011483.1 |
| AL451042.1 |
| AL135924.2 |
| LINC01655 |
| AC022007.1 |
| AL121772.1 |
| AC008083.2 |
| AC110619.1 |
| AC004975.2 |
| MANCR |
| AL121761.1 |
