# Supplementary figures and images for: A stratification system of ferroptosis and iron-metabolism related LncRNAs guides the prediction of the survival of patients with esophageal squamous cell carcinoma
Source: Front Oncol. 2022 Sep 15;12:1010074. doi: 10.3389/fonc.2022.1010074 (PMC9520776; doi:10.3389/fonc.2022.1010074)

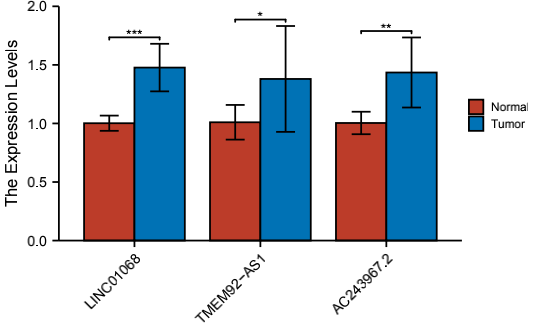

Supplement: Supplementary Figure 1 — The expression of 3 lncRNAs between tumor tissues and normal esophageal tissues. [file Image_1.tif]
